# Exosomes secreted by human urine-derived stem cells could prevent kidney complications from type I diabetes in rats

**DOI:** 10.1186/s13287-016-0287-2

**Published:** 2016-02-06

**Authors:** Zhen-zhen Jiang, Yu-mei Liu, Xin Niu, Jian-yong Yin, Bin Hu, Shang-chun Guo, Ying Fan, Yang Wang, Nian-song Wang

**Affiliations:** Department of Nephrology and Rheumatology, Shanghai Jiao Tong University Affiliated Sixth People’s Hospital, Shanghai, 200233 P.R. China; Institute of Microsurgery on Extremities, Shanghai Jiao Tong University Affiliated Sixth People’s Hospital, Shanghai, 200233 P.R. China

**Keywords:** Human urine-derived stem cells, Exosomes, Diabetes, Podocytes, Apoptosis, Angiogenesis factors

## Abstract

**Background:**

Diabetic nephropathy is one of the most serious complications in patients with diabetes. At present, there are no satisfactory treatments available for diabetic nephropathy. Stem cells are currently the main candidates for the development of new treatments for diabetic nephropathy, as they may exert their therapeutic effects mainly through paracrine mechanisms. Exosomes derived from stem cells have been reported to play an important role in kidney injury. In this article, we try to investigate whether exosomes retrieved from urine stem cells could itself prevent diabetic nephropathy at an early stage in vivo and in vitro.

**Methods:**

Exosomes from conditioned medium of urine-derived stem cells (USCs-Exo) were isolated using ultrafiltration-combined purification methods. USCs-Exo were then verified by morphology, size, and specific biomarkers using transmission electron microscopy, tunable resistive pulse sensing analysis, and western blotting. After establishment of the streptozotocin-induced Sprague–Dawley rat model, the effects of USCs-Exo on kidney injury and angiogenesis were observed via weekly tail intravenous injection of USCs-Exo or control until 12 weeks. In vitro, podocytes cultured in high-glucose medium were treated with USCs-Exo to test the protective effect of USCs-Exo on podocytic apoptosis. Meanwhile, the potential factors in promoting vascular regeneration in USCs-Exo and urine-derived stem cell conditioned medium were investigated by enzyme-linked immunosorbent assay.

**Results:**

Urine-derived stem cells were cultured and were verified by positive markers for CD29, CD73, CD90 and CD44 antigens, and negative markers for CD34, CD45 and HLA-DR. USCs-Exo were approximately 50–100 nm spherical vesicles, and the specific markers included CD9, CD63 and CD81. Intravenous injections of USCs-Exo could potentially reduce the urine volume and urinary microalbumin excretion, prevent podocyte and tubular epithelial cell apoptosis, suppress the caspase-3 overexpression and increase glomerular endothelial cell proliferation in diabetic rats. In addition, USCs-Exo could reduce podocytic apoptosis induced by high glucose in vitro. USCs-Exo contained the potential factors, including growth factor, transforming growth factor-β1, angiogenin and bone morphogenetic protein-7, which may be related with vascular regeneration and cell survival.

**Conclusion:**

USCs-Exo may have the potential to prevent kidney injury from diabetes by inhibiting podocyte apoptosis and promoting vascular regeneration and cell survival.

**Electronic supplementary material:**

The online version of this article (doi:10.1186/s13287-016-0287-2) contains supplementary material, which is available to authorized users.

## Background

Diabetic nephropathy (DN) is one of the most common and serious complications of capillaries in patients with diabetes. At the time of writing, DN affects 20–40 % of patients with diabetes mellitus [1, 2]. There is no satisfactory method or target for the treatment of DN. Hyperglycemia-induced damage to the glomerular podocyte—the terminally differentiated epithelial cells attached to the glomerular basement membrane—is thought to be a critical early event in DN. High glucose contributes to reduced podocyte numbers, induces apoptosis of cultured podocytes, causes albuminuria and accelerates foot process effacement [3]. Interventions that prevent podocyte damage or loss have been demonstrated to have potential for the treatment of DN [4].

DN is also characterized by abnormal angiogenesis driven by several factors, including hyperglycemia and ischemia [5, 6]. Vascular endothelial cells and podocytes act as the barrier to macromolecular flow into the urinary filtrate. The endothelial–podocytes participate in the glomerular filtration barrier, whereby the actions of one type of cell may profoundly influence the function of the other [7]. Interventions that prevent podocyte damage or loss and promote angiogenesis are potential treatments for DN. Bone marrow-derived stem cells as well as mesenchymal stem cells (MSCs) were envisioned for the development of this type of treatment. It has been suggested that the transient presence of MSCs within the injured kidneys may provide a paracrine support for the healing process, including the secretion of cytokines, chemokines and growth factors [8, 9].

Stem cell-derived exosomes have been described as a new mechanism of cell-to-cell communication which have emerged as an important paracrine factor [9]. Exosomes are secreted nanovesicles (30–100 nm diameter) formed through the inward budding of multivesicular bodies that exosomes traffic and transfect proteins, mRNAs and micro-RNAs into target cells could induce tubular differentiation of human vascular endothelial cells (HUVECs) in vitro [10, 11]. Furthermore, new data shows that conditioned media from adipocyte-derived MSCs have the potential to protect podocytes from high-glucose-induced damage, which may be related to the epidermal growth factor secreted by exosomes [12, 13].

Human urine-derived stem cells (USCs) can be conveniently obtained through noninvasive methods. Such methods are thought to be a new portal of access into stem cells [14]. Single clones of USCs can expand to yield a large population, and they have the capacity for multipotent differentiation [15].

In the present study, we investigated the therapeutic effects of exosomes from USCs (USCs-Exo) in a streptozotocin (STZ)-induced rat model. We found that intravenous injections of USCs-Exo could reduce the urine volume and urinary microalbumin excretion. Under further analysis, we found that the apoptosis of podocytes and tubular epithelial cells was prevented, the overexpression of caspase-3 was suppressed, and the proliferation of glomerular endothelial cells was increased, which indicated that inhibiting podocyte apoptosis or promoting vascular regeneration may be one means of preventing kidney complications arising from diabetes. In vitro, USCs-Exo could reduce podocytic apoptosis and contained the potential factors which may be related to vascular regeneration and cell survival. To our knowledge, this is the first study to suggest that USCs-Exo could prevent kidney complications from diabetes, which indicates that USCs-Exo can be a novel regulator in DN therapy.

## Methods

### Ethics, consent and permissions

This study was performed in accordance with the principles of the Helsinki Declaration and was approved by the Ethical Review Board of Shanghai Jiao Tong University Affiliated Sixth People’s Hospital. Human urine samples were collected from six healthy men with a median age of 25 years. Written informed consent was obtained from all study participants. Experiments using Sprague–Dawley (SD) rats were approved by the Animal Care and Use Committee of Shanghai Jiao Tong University Affiliated Sixth People’s Hospital.

### Isolation and culture of USCs

USCs were isolated and identified as previously described [16, 17]. Briefly, penicillin and streptomycin were added to the fresh urine samples (200 ml) at the recommended concentrations to minimize contamination. After the urine sample was centrifuged at 400 × g for 10 minutes at room temperature, the supernatant was discarded and the sediment was washed twice with phosphate-buffered saline (PBS). The resulting sediment was re-suspended in Dulbecco’s modified Eagle medium (DMEM) supplemented with 2 % (vol/vol) fetal bovine serum (FBS; Gibco, USA), 10 ng/ml of human epidermal growth factor (hEGF), 2 ng/ml of platelet-derived growth factor (PDGF), 1 ng/ml of transforming growth factor (TGF)-β, 2 ng/ml of basic fibroblast growth factor (bFGF), 0.5 μM hydrocortisone, 25 μg/ml of insulin, 20 μg/ml of transferrin, 549 ng/ml of epinephrine, 50 ng/ml of triiodothyronine (T_3_), L-glu and antibiotics. The cell suspension was plated into gelatin-coated 24-well plates and incubated at 37 °C in a humidified atmosphere with 5 % CO_2_. The medium was changed after 7 days, and the nonadherent cells were removed by thoroughly washing with PBS. Colonies that were derived from single cells were marked. The culture medium was refreshed twice per week. The cells were passaged using 0.25 % trypsin when they reached approximately 80 % confluence.

### Flow cytometry analysis of USCs

Passage 4 USCs were incubated with 3 % bovine serum albumin for 30 minutes to block nonspecific antigens. The cells were then incubated with the following monoclonal antibodies (Becton Dickinson, USA): CD29-PE, CD73-PE, CD90-PE, CD44-FITC, CD45-FITC, CD34-APC and HLA-DR-PE. Nonspecific fluorescence was determined by incubation of similar cell aliquots with isotype-matched monoclonal antibodies (BD Biosciences). The cells were washed to remove unbound antibodies. Surface antigens were analyzed using a Guava easyCyte™ (Millipore, Billerica, MA, USA).

### Isolation and purification of USCs-Exo

USCs-Exo were prepared and purified as previously described. Kosaka et al. [18] and Montecalvo et al. [19] 80–90 % confluent USCs were washed with PBS and cultured for an additional 48 hours at 37 °C and 5 % CO_2_ in the special USC medium with exosome-free FBS. The conditioned medium was collected and centrifuged at 2500 × g for 20 minutes at 4 °C. After the centrifugation, the supernatant was filtered using a 0.22-μm filter sterilize SteritopTM (Millipore, USA) to remove the remaining cells and cellular debris. Then, the supernatant was transferred to an Ultra-clear tube (Millipore, USA) and centrifuged at 100,000 × g for 70 minutes at 4 °C to deposit USCs-Exo, and then the supernatant was decanted. The USCs-Exo pellet was resuspended in 200ul of PBS. In order to further purification, the liquid containing USCs-Exo was laid on top of 30 % sucrose/D_2_O cushion in a sterile Ultra-Clear™ tube (Beckman Coulter, Kraemer Boulevard Brea, CA, USA) and centrifuged at 100,000 × g for 70 minutes at 4 °C (Beckman Coulter, Sorvall, Avanti J-26XP, fixed angle rotor). USCs-Exo was recovered using an 18-G needle and finally dissolved in 200 μl of PBS. USCs-Exo were stored at −80 °C or used for other downstream experiments. USCs-Exo protein contents were determined using the BCA assay following the instructions (Thermo Fisher, USA). The absorbance was read at 562 nm using a Microplate Reader (Bio-Rad Laboratories, USA).

### Transmission electron microscopy of USCs-Exo

The USCs-Exo fraction was assessed by transmission electron microscopy (TEM). The exosome pellets were fixed in 3 % (w/v) glutaraldehyde and 2 % paraformaldehyde in cacodylate buffer, and then loaded to copper grids coated with Formvar. After washing, the grids were contrasted in 2 % uranyl acetate, dried, and then examined by TEM (Morgagni 268D, Philips, Holland).

### Tunable resistive pulse sensing analysis of USCs-Exo

The size distribution and concentration of USCs-Exo were measured by tunable resistive pulse sensing (TRPS) analysis. TRPS measurements were performed using a qNano platform with an NP100-rated nanopore (Izon Science, UK). The membrane was stretched at 43.0 mm. CPC100 particles (Izon Science) were used to calibrate the size and concentration following manufacturer’s instructions. Samples were diluted 1000-fold with 0.22-μm filtered PBS and measured for 3 minutes. Data processing and analysis were carried out on the Izon Control Suite software v2.2 (Izon Science).

### Western blotting analysis of USCs-Exo

The identity of USCs-Exo had been confirmed by the presence of the specific surface proteins CD9, CD63 and CD81 [20]. Briefly, 5× protein-loading buffer was added directly to the USCs-Exo sample and heated at 95 °C for 5 minutes. Next, USCs-Exo protein was loaded and resolved in 12 % sodium dodecyl sulfate/polyacrylamide gel electrophoresis. The protein sample was run at 120 V for 45 minutes and transferred onto polyvinylidene difluoride membranes (Millipore, USA) for 1.5 hours at 100 mA. The presence of CD9, CD63 and CD81 was tested by exposing the membranes to primary rabbit polyclonal anti-CD9 (1:1000), anti-CD63 (1:1000) and anti-CD81 (1:1000) (Abcam, UK). The membranes were washed three times in 1× Tris-HCl buffered saline Tween (TBS and 0.1 % Tween 20; TBST) for 5 minutes, and incubated for 1 hour in TBST containing horseradish peroxidase-conjugated goat anti-rabbit secondary antibody (Abcam). Proteins were detected using enhanced chemiluminescence (Thermo Fisher, USA) and imaged using an Image Quant LAS 4000 mini bio-molecular imager (Bio-Rad, USA).

### Diabetes rat model and injection of USCs-Exo

Diabetes was induced by a single injection of STZ (Sigma, USA) 65 mg/kg administered to male SD rats (200–250 g). Control rats received citrate buffer. Fifty SD rats were used, and ten were used in preliminary experiments. 10 SD rats did not reach the criteria of diabetes and were excluded. Ten were used as a control group and 20 were randomly grouped as the diabetes group and the diabetes treated with USCs-Exo group. Blood glucose was measured from tail vein blood. Diabetes was defined as a random blood glucose reading ≥16.7 mmol/l for three consecutive days after 72 hours of STZ injection. SD rats who met the criteria for diabetes were randomized to be treated or untreated. The rats of the USCs-Exo treatment diabetic group were injected weekly with Exo (100 μg of Exo dissolved in PBS to final volume of 200 μl) via the tail vein [21]. Other diabetic rats (n = 10) were given the same volume of control medium dissolved in PBS to 200 μl. The random blood glucose and body weight of each rat was measured every week. Twenty-four-hour urine specimens were collected from all rats every 4 weeks, and centrifuged at 3000 rpm for 10 minutes at 4 °C. The supernatant was measured for albumin concentration. Results of urinary albumin were normalized to urinary creatinine levels and expressed as urinary albumin-to-creatinine ratio (UACR). Blood samples were collected from all rats every 4 weeks. The supernatant of blood samples from the tail vein was reserved after centrifugation at 3000 rpm for 10 minutes at 4 °C in order to measure biochemical parameters, including blood glucose, blood urea nitrogen, serum creatinine, and finally tested by an automatic biochemistry analyzer (Hitachi Model 7600, Japan). Twelve weeks after the induction of diabetes, rats were sacrificed under chloral hydrate anesthesia, and kidneys were obtained. The left kidneys were divided into two parts. One part was washed by PBS and fixed with 10 % buffered formalin for paraffin production, for the use of TUNEL analysis, periodic acid-schiff (PAS) staining and immunofluorescence. The other part was separated into the cortex and medulla, in which cortex was immediately placed in liquid nitrogen for tissue protein extraction for Western blotting. The right kidneys were dealt with in the same way, and conserved for later use. Glomerular injury was evaluated by mesangial expansion in sections stained with hematoxylin and eosin under light microscopy (200X). The mesangial area was counted as mesangial expansion, which was determined in 20 consecutive glomeruli from each rat. All slides were observed independently by two blinded investigators. Relative mesangial expansion was described as the fold change from the normal control group.

### TUNEL assay

After deparaffinization (using xylene and ethanol dilution) and rehydration the sections were stained for TUNEL Kit (11684817910, Rocha Applied Science, Switzerland) as described in the Kit instructions. Briefly, each slide was deparaffinized and rehydrated, and treated with proteinase K (20 mg/l) for 15 minutes. The endogenous peroxidase was inhibited with 3 % hydrogen peroxide for 5 minutes, and then incubated with the TUNEL reaction mixture containing terminal deoxynucleotidyl transferase (TdT) and digoxigenin-11-dUTP for 1 hour. The TdT reaction was carried out in a humidified chamber at 37 °C, and then 3,3-diaminobenzidine chromogen was applied. Hematoxylin was used as counterstaining. For the negative control, TdT was omitted from the reaction mixture. Apoptotic cell number was quantitatively analyzed by counting the TUNEL-positive cells selected randomly from 10 fields, at 40× magnification. Results were presented as the number of TUNEL-positive cells per 10^3^ cells.

### Analysis of caspase-3 protein expression

Renal cortical protein was separated by dodecyl sulfate/polyacrylamide gel electrophoresis and transferred to a polyvinylidene difluoride membrane. The membranes were then blocked by incubation in TBST containing 5 % bovine serum albumin and incubated overnight at 4 °C with the primary antibody, caspase-3 (ab32351; Abcam, USA). Relative protein expression was described as the fold change from the normal control group.

### Immunofluorescence staining of CD31

The expression of CD31 known as platelet-endothelial cell adhesion molecule-1 was detected by immunofluorescence staining in each group. Briefly, the paraffin sections were placed in an oven at 60 °C for 1 hour and then rehydrated in a graded ethanol series (100 %, 95 %, 90 %, 85 %, and 70 %). Citrate buffer was used for heat-induced antigen retrieval. Then goat serum was used as a blocking solution before the primary antibody was applied. The sections were incubated overnight in 4 °C with rabbit anti-rat CD31 antibody at a 1:150 dilution (Abcam, USA), and mouse anti-rat Ki-67 (a marker of proliferation) antibody at a 1:100 dilution (Abcam, USA).The secondary antibody, goat anti-rabbit antibody conjugated to Alexa Fluor 594 (BA1032, China) and goat anti-mouse antibody conjugated to Alexa Fluor 488 (Life, USA) were applied at 1:200 dilutions for 1 hour. Then immunofluorescence photomicrographs were obtained at 400× magnifications using a fluorescence microscope (Leica, Solms, Germany), and the intensity of the CD31-positive signal was quantified with Image pro plus 6.0 image analysis software (Media Cybernetics, USA) to represent glomerular endothelial cell proliferation.

### PAS staining

The kidneys were stained with PAS. The sections were then examined by light microscopy. Glomerular injury was evaluated by mesangial expansion in sections stained with PAS. Summarily, the mesangial area was counted as mesangial expansion, which was determined in 20 consecutive glomeruli from each rat. Relative mesangial expansion was described as the fold change from the normal control group.

### Podocyte culture with high glucose in vitro

Conditionally immortalized human podocytes were obtained from Dr. Moin Saleem, Bristol, UK, and later donated to us by John Cijiang. Podocytes were cultured as described previously [22]. Briefly, podocyte recovery was at 33 °C, used an RPMI 1640 medium containing 10 % FBS and 1 × ITS culture proliferated. Then, cells were inoculated into different six-well plates and were cultured for at least 7 days as a mature podocyte at 37 °C so they could be used for the tests [23]. The cells were cultured synchronously in medium containing 0.1 % FBS and 5.5 mM D-glucose RPMI-1640 medium for 24 hours before the test. Then they were divided into six groups according to treatment: normal glucose (5.5 mM), mannitol control (5.5 mM D-glucose + 24.5 mM mannitol), high glucose (30 mM), and treatment groups with USCs-Exo (5 μg/ml, 10 μg/ml and 50 μg/ml). Each group was subjected to its own designated treatment regimen for 72 hours.

### Flow cytometry analysis detection of podocytic apoptosis

According to the operation instructions of Annexin V-FITC/PI Apoptosis Detection Kit (Dojindo, Kumamoto, Japan), podocytes (5 × 10^5^/ml) were collected and suspended with 200 μl of binding buffer, and then 10 μl of Annexin-v-FITC and 5 μl of PI were added for a 15 minute light cycle avoiding incubation at room temperature. Lastly, the cells were resuspended in 250 μl of binding buffer and analyzed using the guava easyCyteTM system (Millipore, Billerica, MA, USA). The experiment had three replicates.

### Cytokines in USCs-Exo detected by enzyme-linked immunosorbent assay

The concentrations of vascular endothelial growth factor (VEGF), TGF-β1, angiogenin and bone morphogenetic protein (BMP)-7 were determined in USCs-Exo and USC conditioned medium (USC-CM) according to the manufacturer’s instructions. Primitive USC medium was taken as control. Optical density (OD) was measured at 450 nm. The concentration of candidate factors in the samples was calculated according to standard curves. Three independent samples were placed in three repetitive holes.

### Statistical analysis

The data are listed as means ± standard deviation. Statistical analyses were carried out using GraphPad Prism 5.0. Two-way analysis of variance (ANOVA) or one-way ANOVA was used to compare differences in outcome variables between different groups. Subgroup analysis was performed by S-N-K test. A result was considered statistically significant at *P* < 0.05.

## Results

### USC phenotype

**Fig. 1:**
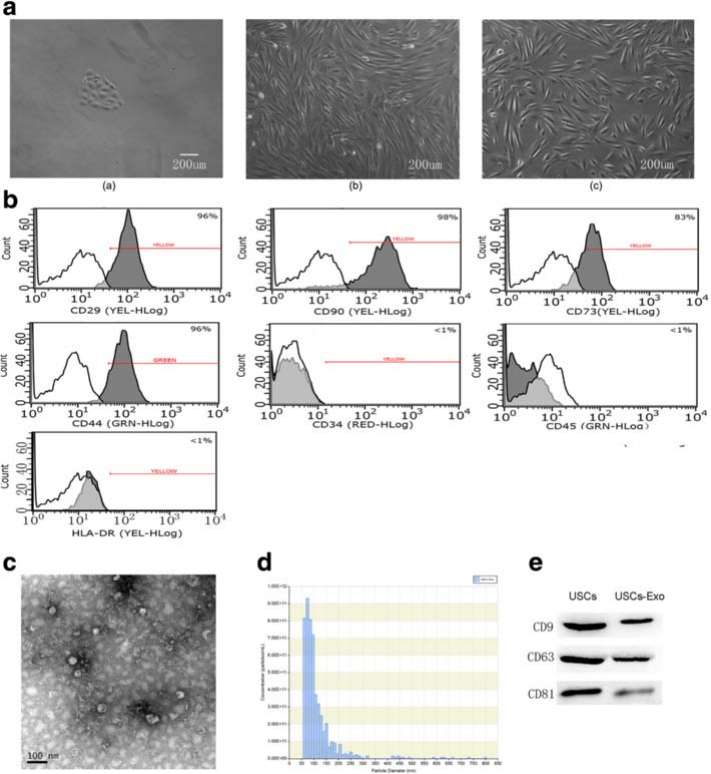
Characteristics of USCs and USCs-Exo. **a**) The morphology and growth of USCs. Scale bar = 200 μm. **b**) USCs were characterized by flow cytometry using the surface markers CD29, CD90, CD73, CD44, CD34, CD45 and HLA-DR. *White* solid peaks represent the isotype controls and the *grey* solid peak represents the marker indicated. **c**) Morphology of USCs-Exo under a transmission electron microscopy. **d**) TRPS measurement showed that the size range of USCs-Exo concentrated at 50–100 nm, and the measured mean concentration (particles/ml) of USCs-Exo was 5.2E + 009. **e**) Western blotting analysis of exosome-specific CD9, CD63 and CD81 proteins in USCs and USCs-Exo. *USC* Urine-derived stem cell, *USCs-Exo* Exosome from urine-derived stem cells

Cell colonies were observed in the USC-cultured plates approximately 7 to 10 days after initial plating. USCs were confirmed using light microscopy to verify a fibroblast-like morphology (Fig. 1a, left panel). The fibroblast-like cells had a robust proliferation capability and reached 80–90 % confluence after 16 days (Fig. 1a, middle panel). After several passages, USCs retained the elongated morphology (Fig. 1a, right panel). As the criterion to identify stem cells, cell surface markers must be analyzed. We used flow cytometry analysis (FCM) to detect the surface antigen expression. The results demonstrated that the USCs were positive for CD29, CD73, CD90 and CD44 antigen and negative for CD34, CD45 and HLA-DR (Fig. 1b). When cultured in osteogenic, adipogenic, or chondrogenic media, the USCs could differentiate into osteoblasts, adipocytes and chondrocytes as previously reported by the authors [16, 17]. Thus, the characterization of USCs meets the criteria for defining multipotent MSCs.

### Characterization of USCs-Exo

To investigate the roles of USCs-Exo in diabetic rats, USCs-Exo were extracted and identified. The morphology of USCs-Exo was observed under TEM, and their size was measured by Nano Sight analysis. The results of TEM showed that USCs-Exo were spherical vesicles of about 100 nm (Fig. 1c). TRPS analysis showed that the size of USCs-Exo were approximately 50–100 nm (Fig. 1d), which was in accord with TEM. The results of Western blotting showed that exosomes markers, including CD9, CD63 and CD81, were expressed in USCs-Exo (Fig. 1e).

### Intravenous injection of USCs-Exo could reduce the urine volume and urinary microalbumin excretion of diabetic rats

**Fig. 2:**
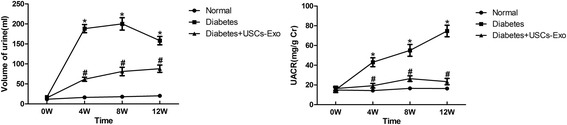
Intravenous injection of USCs-Exo could reduce the urine volume and urinary microalbumin excretion in type 1 diabetic rats. Changes in urinary volume and urinary albumin to creatinine ratio (*UACR*) in different groups were analyzed. Values are shown as the mean ± standard deviation for the groups normal (n = 10), diabetes (n = 10), and diabetic rats treated with USCs-Exo (n = 10). *P < 0.05, versus normal; ^#^ *P* < 0.05, versus diabetic rats + USCs-Exo. *Cr* Creatinine, *USCs-Exo* Exosome from urine-derived stem cells, *W* Weeks

We established the rat model of DN induced by intraperitoneal injection of STZ to test the hypothesis that USCs-Exo has some beneficial effects on the kidney in diabetic rats. The results showed that polyuria was evidently improved in the diabetes treated with USCs-Exo group compared with the diabetes only group (Fig. 2, left panel). To evaluate the level of microalbuminuria in different groups, urinary albumin concentration was expressed as UACR. Compared with the normal group, the rats in the diabetes model group showed a marked elevation of UACR (Fig. 2, right panel). USCs-Exo treatment significantly suppressed UACR of diabetes rats at every time point (Fig. 2, right panel). Blood glucose was significantly increased in STZ-induced diabetic rats when compared with normal control rats. However, no differences in blood glucose, serum creatinine or blood urea nitrogen were observed between USCs-Exo treated and untreated diabetic rats (Additional file 1: Table S1). These results suggest that USCs-Exo may play an important role in preventing renal function decline in type 1 diabetic rats.

### Intravenous injection of USCs-Exo could prevent cells apoptosis and suppressed caspase-3 overexpression in diabetic rats

**Fig. 3:**
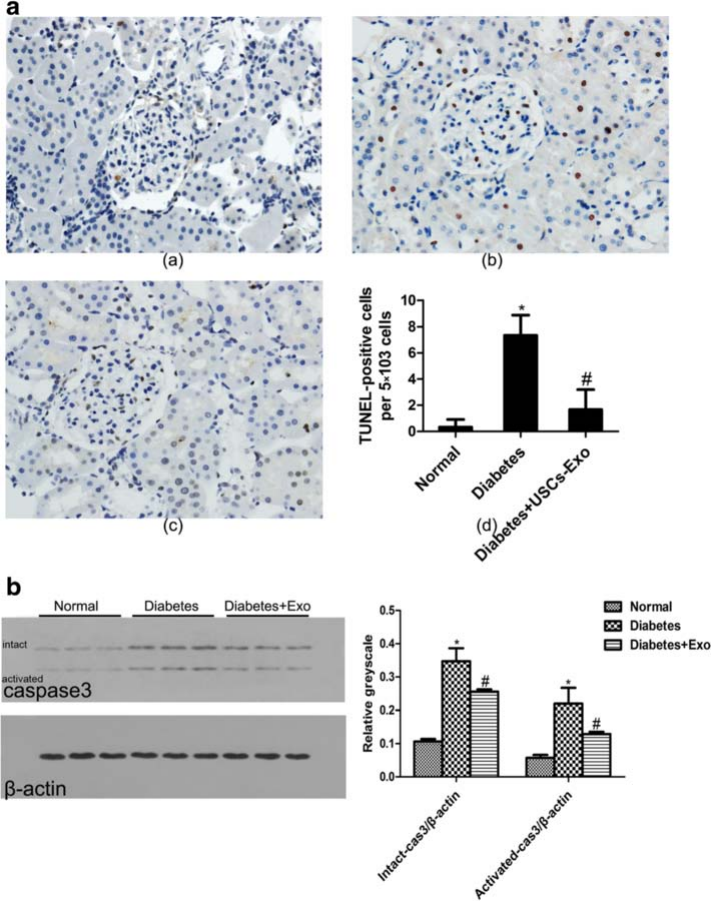
Intravenous injection of USCs-Exo could attenuate the expression of apoptosis-related protein and TUNEL-positive cells. **a**) Tissue apoptosis was examined with TUNEL-positive staining; (*a*) normal, (*b*) diabetes, (*c*) diabetes treated with USCs-Exo and (*d*) semi-quantitative analysis for apoptotic examination was scored. **b**) The relative protein levels of activated caspase-3/β-actin and intact caspase-3/β-actin was detected by Western blotting. *P < 0.05, versus normal; ^#^ *P* < 0.05, versus diabetes + USCs-Exo. *USCs-Exo* Exosome from urine-derived stem cells

We assessed podocyte and tubular epithelial cell apoptosis in diabetic rats by TUNEL staining and further tested caspase-3, an apoptosis-related protein. There was a significant decrease in apoptotic cells in kidney sections from diabetes treatment with USCs-Exo (*P* < 0.05) (Fig. 3a). TUNEL assay showed some TUNEL-positive cells in the glomerular and tubule region in diabetic rats (Fig. 3a*b*). However, the apoptosis-positive cells in the normal control group (Fig. 3a*a*) and diabetes treatment with USCs-Exo rats (Fig. 3a*c*) were very few. Following on from this, Western blotting was used to measure caspase-3, an apoptosis-related protein. We observed that the relative activated caspase-3 protein (activated-cas3/β-actin) and intact caspase-3 (intact-cas3/β-actin) protein levels in diabetic rats were higher than those in the normal group (*P* < 0.05) (Fig. 3b). Then, compared with the diabetic group, USCs-Exo downregulated caspase-3 protein overexpression (*P* < 0.05) (Fig. 3b).

### Intravenous injection of USCs-Exo could promote glomerular endothelial cell proliferation in the early stage of diabetic kidney impairment

**Fig. 4:**
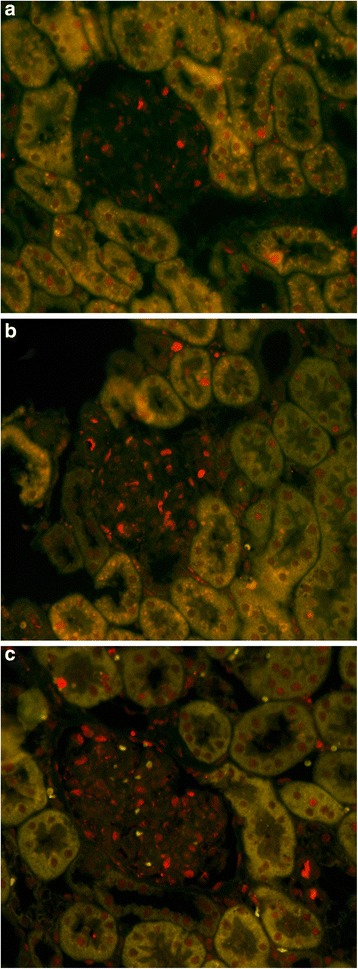
Double immunofluorescent staining of CD31 (*red*) and Ki-67 (*green*). **a**) normal group, **b**) diabetes group, and **c**) diabetes treated with USCs-Exo group. Representative photomicrographs of the designated area

As we know, the glomerular filtration barrier is composed of endothelial cells, basement membrane and podocytes. The loss of glomerular capillaries plays an important role in albuminuria production and renal function decline. The results of immunofluorescence co-stain with CD31 and Ki-67 showed that endothelial cells had obvious proliferation. The amount of CD31- and Ki-67-positive endothelial area was moderately increased in the diabetes + USCs-Exo group (Fig. 4c), but there were no obvious Ki-67-positive areas in the normal group (Fig. 4a) or diabetes group (Fig. 4b).

### Intravenous injection of USCs-Exo ameliorated the renal histopathology

**Fig. 5:**
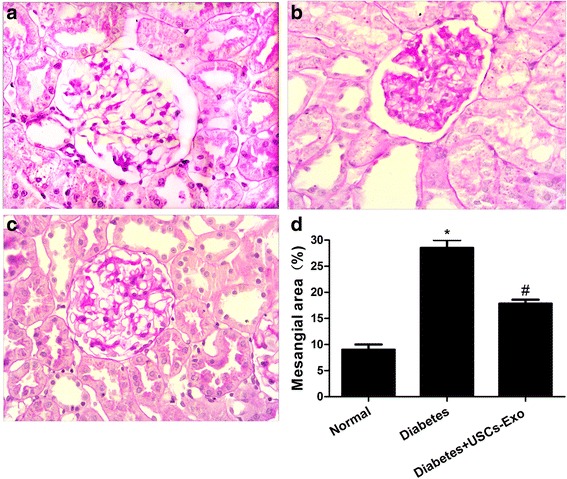
Intravenous injection of USCs-Exo could ameliorate the renal histopathology changes in diabetic rats at 12 weeks. Representative light microscope appearance of PAS staining in glomeruli. (**a**) normal, (**b**) diabetes, and (**c**) diabetes treated with USCs-Exo. *Purple-red* represents a positive region, suggesting mesangial matrix proliferation. (**d**) Quantitative analysis of mean mesangial area from each group of rats. Magnification 400×. Results are expressed as the means ± standard deviation. *P < 0.05, versus normal; ^#^ *P* < 0.05, versus diabetes. *USCs-Exo* Exosome from urine-derived stem cells

Twelve weeks after the establishment of the diabetes model, the diabetic rats showed focal mesangial matrix expansion compared to normal rats (Fig. 5a). Kidney sections from diabetic rats revealed a remarkable glomerular hypertrophy and increased intraglomerular cells mostly in the mesangial area with mesangial expansion (Fig. 5b). In diabetic rats with USCs-Exo, however, there were only modest increases in the mesangial matrix and intraglomerular cells, with no apparent signs of glomerular hypertrophy and mesangial matrix proliferation (Fig. 5c). The results of quantitative analysis showed that USCs-Exo treatment significantly ameliorated mesangial expansion (*P* < 0.05) (Fig. 5d).

### USCs-Exo could reduce podocytic apoptosis induced by high glucose in vitro

**Fig. 6:**
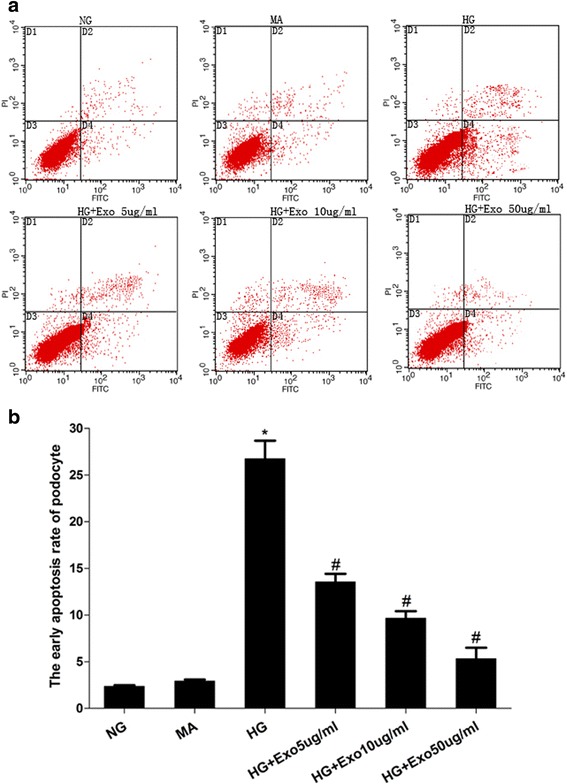
USCs-Exo could reduce apoptosis of human podocyte cells induced by high glucose (*HG*). **a**) Representative FCM photograph of PI and AnnexinV-FITC double stained podocyte apoptosis. **b**) The bar chart of PI and AnnexinV-FITC double stained podocyte apoptosis. *P < 0.05, HG group versus normal glucose (*NG*) group; ^#^ *P* < 0.05, HG group versus HG treated with USCs-Exo group. *MA* Mannitol

To define the role of USCs-Exo in changes to cell apoptosis in rats, we added USCs-Exo to human podocytes exposed to high glucose for 72 hours in vitro. The number of apoptotic or necrotic cells was quantified by FCM analysis after staining with Annexin V and PI (Fig. 6a). The cytograms show viable cells that did not bind Annexin V or PI in the D3 quadrant. Cells in the early stages of apoptosis that bound Annexin V but still had intact cell membranes and excluded PI are shown in the D4 quadrant. Cells with advanced stages of apoptosis or necrosis that were both Annexin V- and PI-positive are shown in the D2 quadrant. Cells that lost their intact cell membranes that bound PI and excluded Annexin V are shown in the D1 quadrant. The results are presented in the form of a percentage, and showed that the early podocytic apoptosis rate in the D4 quadrant was significantly higher in the high glucose group than in normal glucose group (26.73 versus 2.36 %), and USCs-Exo-reduced podocytic apoptosis was induced by high glucose in a dose-dependent manner (13.5 versus 9.7 versus 5.2 %; *P* < 0.05) (Fig. 6b).

### USCs-Exo may contain the potential factors for promoting angiogenesis and cell survival

**Fig. 7:**
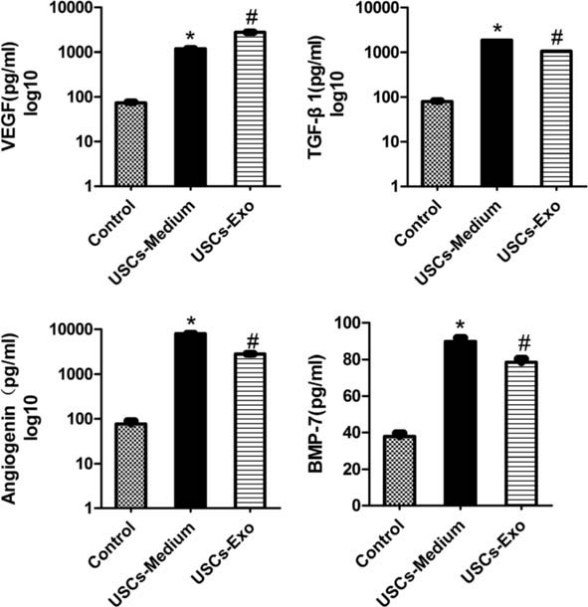
ELISA assay of USC-CM and USCs-Exo for potential factors. The concentrations of VEGF, TGF-β, angiogenin and BMP-7 in control, USC-CM and USCs-Exo were measured by ELISA. *P < 0.05, USC-CM versus control; ^#^ *P* < 0.05, USCs-Exo versus control. *BMP-7* Bone morphogenetic protein-7, *TGF-β1* Transforming growth factor-β1, *USC* Urine-derived stem cell, *VEGF* Vascular endothelial growth factor

To investigate the potential mechanisms by which USCs-Exo inhibited podocytic apoptosis, increased glomerular endothelial cells and promoted glomerular angiogenesis, we performed enzyme-linked immunosorbent assay (ELISA) to determine whether podocyte survival factor (BMP-7) and angiogenesis related proteins (VEGF, TGF-β1 and angiogenin) were present in USCs-Exo [13, 24]. The results demonstrated that VEGF, TGF-β1, angiogenin and BMP-7 were significantly higher in USC-CM and USCs-Exo than controls (*P* < 0.05) (Fig. 7). In addition, there were no significant differences between USCs-Exo and USC-CM. Based on these data, we all but confirmed that USCs secreted exosomes carrying some BMP-7 and high levels of VEGF, TGF-β and angiogenin to be involved in renal protection in diabetes.

## Discussion

In this study, we obtained human-derived stem cells and tested their multipotent capacity. We successfully obtained USCs-Exo and investigated their role in diabetic rats. USCs-Exo treatment significantly suppressed UACR in diabetic rats. Intravenous injection of USCs-Exo could prevent podocyte apoptosis by suppressing caspase-3 overexpression and increasing glomerular endothelial cell proliferation in the early stage of diabetes in the test subjects. USCs-Exo could reduce podocytic apoptosis induced by high glucose in vitro. USCs-Exo and USC-CM contained the potential factors (VEGF, TGF-β1, angiogenin and BMP-7) for promoting vascular regeneration and cell survival.

DN is the leading cause of end-stage renal disease and is defined by pathological changes in the kidney causing progressive loss of glomerular filtration rate, proteinuria and tubulointerstitial fibrosis. Treatments currently available include tight glucose and blood pressure control and blockade of the renin–angiotensin system, which can delay, but not prevent, the development of DN. Consequentially, the need to develop new therapies to target DN is essential. Several factors have been shown to influence the progression of DN after the onset of albuminuria; TGF-β, angiotensin II, and advanced glycated proteins have been extensively characterized [4]. Furthermore, podocytes are highly differentiated cells that play an important role in the pathogenesis of DN [25]. Our study was carried out based on these mechanisms behind DN. Podocytes and endothelial cells mainly take part in the formation of the filtration barrier. Glucose at high levels could reduce the number and density of podocytes, destroy the integrity of the glomerular filtration membrane and change its selective permeability, so as to promote the progression of glomerular sclerosis and accelerate the progression of DN. Meanwhile, the loss and injury of interacting proteins of podocytes could activate apoptosis and destroy the integrity of the slit membrane, which in turn aggravates proteinuria and accelerates DN progression [4, 26, 27]. On the other hand, angiogenesis is the formation of new vascular segments originating from existing vessels, which is characterized by a combination of sprouting of new vessels from the sides and ends of pre-existing ones or by longitudinal division of existing vessels with periendothelial cells. As there is proven interaction between podocytes and endothelial cells, angiogenesis might be related to the mechanism of DN. Angiogenesis requires many interactions that must be regulated by a wide range of angiogenic inducers, including growth factors, chemokines, angiogenic enzymes, endothelial-specific receptors, and adhesion molecules, as well as various endogenous angiogenesis inhibitors [6, 28].

Stem cell therapies have been studied in several kidney injury models. Many studies indicated that it is the paracrine pathway rather than a direct effect responsible for stem cell damage repair. It is likely that a large complex rather than a single small molecule (believed to be microvesicles), or the exosomes, are responsible for the paracrine pathway [29–31]. Exosomes were first found to be secreted by sheep reticulocytes approximately 50 years ago. They have since been shown to be secreted by many cell types, including B cells, dendritic cells, mast cells, platelets, and so forth. They are also found in physiological fluids such as normal urine, plasma and bronchial lavage fluid. They have a diameter of 40–100 nm, with a density in sucrose of 1.13–1.19 g/ml. Most exosomes have an evolutionarily conserved set of proteins, including tetraspanins (CD81, CD63 and CD9), Alix and Tsg101, yet they also have unique tissue/cell type-specific proteins that reflect their cellular source. The large diversity in exosome-secreting cell types and the presence of exosomes in different physiological fluids indicate that secretion of exosomes is a common cellular function. They are believed to be important for intercellular communications [32]. Exosomes may act as important paracrine/endocrine mediated factors, which can transfer specific protein, mRNA and microRNA. Unlike cells, exosomes do not elicit acute immune rejection and, being nonviable and much smaller, they pose less safety risks such as the formation of tumors or embolisms, which is thought to be a novel vehicle in medical engineering [33]. However, whether exosomes could access to podocytes or endothelial cells need to be further studied to demonstrate a USCs exosomes - podocyte interaction in vivo.

Exosomes have been studied in many acute kidney injury (AKI) models such as ischemia–reperfusion injury (IRI) [34], AKI [35], cisplatin-treated model [36], and so forth. Previous studies indicated that, in a lethal mouse AKI model, multiple injections of exosomes could decrease the mortality of AKI mice. The mechanism of protection was mainly ascribed to an antiapoptotic effect of exosomes [37, 38]. In vitro, studies demonstrated that exosomes upregulated cisplatin-treated human tubular epithelial cell antiapoptotic genes, such as Bcl-xl, Bcl2 and BIRC8, and downregulated genes that have a central role in the execution-phase of cell apoptosis such as Casp1, Casp8 and LTA [21]. Cantaluppi et al. previously found that in an IRI model that microvesicles released from progenitor cells activated an angiogenic program in endothelial cells by horizontal mRNA transfer. The RNA content of microvesicles was enriched in microRNAs that modulate proliferation, angiogenesis, and apoptosis [39]. Recently, many studies had confirmed that exosomes could promote angiogenesis and inhibit apoptosis [40–42]. We have investigated whether USCs-Exo promote endothelial cell proliferation by immunofluorescence of CD31 and Ki-67. The results showed some endothelial areas with CD31- and Ki-67-positive cells and confirmed glomerular angiogenesis. Based on previous studies, we proposed that exosomes derived from USCs may have the ability to prevent kidney impairment in diabetes. Our study indicated that injection of USCs-Exo could reduce podocytic apoptosis by suppressing overexpression of caspase-3 and promoting vascular regeneration and cell survival, which may be related with the potential cytokines VEGF, TGF-β1, angiogenin and BMP-7 contained in USCs-Exo. Podocyte apoptosis was inhibited, associated with BMP-7. Glomerular endothelial cell proliferation and glomerular angiogenesis was increased in diabetic rats due to VEGF, TGF-β and angiogenin [13, 43, 44]. The expression of cytokines such as VEGF or BMP-7 is important for podocyte and endothelial cell survival. However, further studies, especially with regard to the mechanisms and pathways of exosome actions on podocytes or endothelial cells, are to be conducted.

Exosomes have been studied less in chronic damage models; however, microvesicles or the exosomes could also protect against the progression of chronic kidney damage by inhibiting capillary rarefaction, glomerulosclerosis, and tubulointerstitial fibrosis. It has been studied in a 6-month after IRI kidney model that rarefaction of renal microvascular density in the presence of sustained hypoxia is associated with an accelerated progression toward chronic kidney disease. The microvesicles could significantly reduce glomerulosclerosis, tubulointerstitial fibrosis, and microvascular rarefaction, thus preserving renal function [45]. Our study first indicated that exosomes could prevent kidney impairment at the early stage in a diabetic model. The capacity of exosomes has made it increasingly attractive as a potential treatment of DN.

Compared with other stem cells, USCs have their own advantages, especially in urinary regenerative engineering. USCs have stem cell characteristics with a highly proliferative capacity because of their expression of telomerase activity. USCs have more homology with the urinary system, though the origin of USCs is still a matter of debate. They can give rise to additional specialized cell types, including osteocytes, chondrocytes, myocytes and adipocytes. They can be easily and consistently obtained by a noninvasive approach and extensively expanded in vitro so that it is so easy to obtain sufficient cells for use [46, 47]. Combining the advantages of USCs and exosomes, USC-Exo will be a promising therapeutic approach in regenerative medicine, with less immune rejection, better differentiation and a more stable and adequate supply.

## Conclusions

In conclusion, exosomes extracted from human urine-derived stem cells could ameliorate kidney impairment in type I diabetic rats. Exosomes may exhibit their effect by inhibiting podocyte apoptosis or promoting angiogenesis and cell survival through potential factors. Overall, the application of USCs-Exo may be a novel therapeutic approach in the treatment of DN.

## Additional file

Additional file 1: Table S1. Blood glucose, blood urea nitrogen and serum creatinine in rats by groups. (DOCX 181 kb)

## Abbreviations

AKI: Acute kidney injury
ANOVA: Analysis of variance
bFGF: Basic fibroblast growth factor
BMP: Bone morphogenetic protein
CM: Conditioned medium
DMEM: Dulbecco’s modified Eagle medium
DN: Diabetic nephropathy
ELISA: Enzyme-linked immunosorbent assay
FBS: Fetal bovine serum
FCM: Flow cytometry analysis
hEGF: Human epidermal growth factor
HUVEC: Human vascular endothelial cell
IRI: Ischemia–reperfusion injury
MSC: Mesenchymal stem cell
OD: Optical density
PAS: Periodic acid-schiff
PBS: Phosphate-buffered saline
PDGF: Platelet-derived growth factor
SD: Sprague–Dawley
STZ: Streptozotocin
T_3_: Triiodothyronine
TBST: Tris-HCl bufferred saline and Tween mixture
TdT: Terminal deoxynucleotidyl transferase
TEM: Transmission electron microscopy
TGF: Transforming growth factor
TRPS: Tunable resistive pulse sensing
UACR: Urinary albumin-to-creatinine ratio
USC: Urine-derived stem cell
USCs-Exo: Exosome from urine-derived stem cells
VEGF: Vascular endothelial growth factor
