# Supplementary material for: Exosomes secreted by human urine-derived stem cells could prevent kidney complications from type I diabetes in rats
Source: Stem Cell Res Ther. 2016 Feb 6;7:24. doi: 10.1186/s13287-016-0287-2 (PMC4744390; doi:10.1186/s13287-016-0287-2)
Supplement: Additional file 1: Table S1. — Blood glucose, blood urea nitrogen and serum creatinine in rats by groups. (DOCX 181 kb) [file 13287_2016_287_MOESM1_ESM.docx]

**Additional file 1**

Table S1 Blood glucose, blood urea nitrogen and serum creatinine in rats by groups


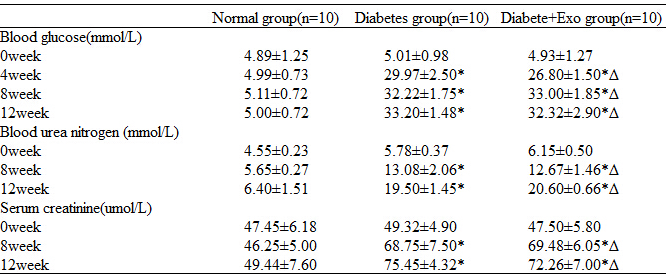


Table S1. No differences in blood glucose, serum creatinine and blood urea nitrogen were observed between USCs-Exo treated and untreated diabetic rats (*P<0.05 vs. normal group; ΔP>0.05 vs. diabetes group).
